# Impact of breeding for reduced methane emissions in New Zealand sheep on maternal and health traits

**DOI:** 10.3389/fgene.2022.910413

**Published:** 2022-09-30

**Authors:** Sharon M. Hickey, Wendy E. Bain, Timothy P. Bilton, Gordon J. Greer, Sara Elmes, Brooke Bryson, Cesar S. Pinares-Patiño, Janine Wing, Arjan Jonker, Emily A. Young, Kevin Knowler, Natalie K. Pickering, Ken G. Dodds, Peter H. Janssen, John C. McEwan, Suzanne J. Rowe

**Affiliations:** ^1^ Ruakura Research Centre, AgResearch Ltd., Hamilton, New Zealand; ^2^ Invermay Agricultural Centre, AgResearch Ltd., Mosgiel, New Zealand; ^3^ Grasslands Research Centre, AgResearch Ltd., Palmerston North, New Zealand

**Keywords:** methane emissions, sheep, parasites, genetic correlation, ruminant, wool, survival, rumen

## Abstract

Enteric methane emissions from ruminants account for ∼35% of New Zealand’s greenhouse gas emissions. This poses a significant threat to the pastoral sector. Breeding has been shown to successfully lower methane emissions, and genomic prediction for lowered methane emissions has been introduced at the national level. The long-term genetic impacts of including low methane in ruminant breeding programs, however, are unknown. The success of the New Zealand sheep industry is currently heavily reliant on the prolificacy, fecundity and survival of adult ewes. The objective of this study was to determine genetic and phenotypic correlations between adult maternal ewe traits (live weight, body condition score, number of lambs born, litter survival to weaning, pregnancy scanning and fleece weight), faecal and *Nematodirus* egg counts and measures of methane in respiration chambers. More than 9,000 records for methane from over 2,200 sheep measured in respiration chambers were collected over 10 years. Sheep were fed on a restricted diet calculated as approximately twice the maintenance. Methane measures were converted to absolute daily emissions of methane measured in g per day (CH_4_/day). Two measures of methane yield were recorded: the ratio of CH_4_ to dry matter intake (g CH_4_/kg DMI; CH_4_/DMI) and the ratio of CH_4_ to total gas emissions (CH_4_/(CH_4_ + CO_2_)). Ewes were maintained in the flocks for at least two parities. Non-methane trait data from over 8,000 female relatives were collated to estimate genetic correlations. Results suggest that breeding for low CH_4_/DMI is unlikely to negatively affect faecal egg counts, adult ewe fertility and litter survival traits, with no evidence for significant genetic correlations. Fleece weight was unfavourably (favourably) correlated with CH_4_/DMI (r_g_ = −0.21 ± 0.09). Live weight (r_g_ = 0.3 ± 0.1) and body condition score (r_g_ = 0.2 ± 0.1) were positively correlated with methane yield. Comparing the two estimates of methane yield, CH_4_/DMI had lower heritability and repeatability. However, correlations of both measures with adult ewe traits were similar. This suggests that breeding is a suitable mitigation strategy for lowering methane yield, but wool, live weight and fat deposition traits may be affected over time and should be monitored.

## Introduction

New Zealand is heavily reliant on pastoral-based agriculture and has a national sheep population of ∼17 million breeding ewes (Beef and Lamb NZ Economic reports, 2020). Maternal sheep production in New Zealand relies on prolificacy, fecundity and survival of adult ewes. Sheep breeders can obtain breeding values for their stock (Newman et al., 2009), expressed as NZ$ gross profit per breeding ewe. The sustainability and therefore profitability of this system, however, is facing a new threat as awareness grows of the magnitude and impact of ruminant methane emissions on the environment. Ruminant enteric methane emissions are responsible for 35% of New Zealand’s total greenhouse gas emissions and account for 73% of all New Zealand’s agricultural emissions, with 30% of enteric methane directly attributable to the sheep industry (Ministry for the Environment, 2021). International commitments to reduce methane emissions to mitigate the impacts of climate change have been made by over 50 countries, and the recent Global Methane Pledge is to lower the 2020 methane emissions level by 30% by 2030, in line with the UNFCCC (2015). Strategies, such as carbon taxes on livestock production, are being developed to protect the environment and maintain global food security (Golub et al., 2013; Springmann et al., 2016). The majority of enteric methane emissions from sheep production can be attributed to the ewe flock, which is maintained throughout the entire year, including the winter months when feed quality is low. Independent breeding strategies exist for increased maternal production and reduced methane emissions but, to date, there are no data to show whether these breeding objectives might be synergistic, neutral or antagonistic.

Breeding for lowered methane emissions has been shown as a permanent and cumulative strategy for the mitigation of methane in sheep (Rowe et al., 2019). The heritability of absolute methane emissions in sheep has been shown to range between 0.13 (Goopy et al., 2015) and 0.29 ± 0.05 (Pinares-Patiño et al., 2013), with the heritability of methane yield expressed as methane emissions per kg dry matter intake (DMI) of 0.13 ± 0.03 (Pinares-Patiño et al., 2013). Selection lines based on methane emissions per kg DMI are maintained in New Zealand (Pinares-Patiño et al., 2013; Rowe et al., 2019). After two generations of selection for either high or lower methane yield, the low line averages a 12% lower methane yield than the high line (Rowe et al., 2019). Using 1-hour measures in portable accumulation chambers as an alternative to full respiration chamber measures (Jonker et al., 2018), breeders can use a mixture of short-term measures and genomic prediction to rank individual animals and select parents for lowered methane emissions. Rowe et al. (2021) have shown that incorporating methane emissions into the national breeding scheme is an extremely low-cost and effective mitigation strategy for lowering the greenhouse gas emissions from sheep. Before this strategy is implemented on a national scale, however, breeders must understand the long-term consequences of introducing methane emission traits. Animals with lower methane emissions have physiological differences from their high-emitting counterparts. These include 20% smaller rumens (Goopy et al., 2013, 2014; Bain et al., 2014), different microbial fermentation profiles (Kittelmann et al., 2014; Hess et al., 2020) and a higher ratio of propionate to butyrate in the volatile fatty acids coming from the rumen used by the animal as an energy source (Pinares-Patiño et al., 2011; Jonker et al., 2018). There is preliminary evidence that these changes are also associated with a leaner animal (Elmes et al., 2014).

Methane emissions are mainly measured in young animals (5–10 months of age). Therefore, methane parameters need to be validated in adult animals, particularly adult ewes, as they are maintained in the flock for several years. If low-emitting animals do have less fat and greater lean tissue, as suggested by Elmes et al. (2014), it could be hypothesized that this might, whilst positively affecting carcass value, have a negative effect on the ability of ewes to overwinter and to maintain productivity. Adult ewe body condition is thought to be a trait associated with ewe longevity, lamb survival and growth and the ability to survive feed fluctuations, often encountered in hilly environments (Morgan-Davies et al., 2007; Everett-Hincks and Dodds, 2008; Borg et al., 2009).

The ewe’s ability to remain healthy and in good condition through winter and then to mother and raise lambs is fundamental to production. A great health challenge is a parasitism from intestinal nematodes. There has been some evidence that the parasite burden is correlated with methane emissions (Fox et al., 2018). In addition to adult ewe traits, we investigated relationships between methane emissions and faecal egg counts of Strongyle (FEC1) and *Nematodirus* spp. egg counts (NEM1) in summer at approximately 5 months of age.

Relationships between methane emissions and adult ewe traits associated with health, prolificacy, fecundity and survival, commonly described as “maternal traits”, have not previously been investigated. Given the long-term impact of sheep production on methane emissions will come predominately from the adult breeding ewe, it is vital that the relationship between methane and maternal traits is assessed and that methane parameters measured in young animals are validated in adults. Furthermore, maternal traits have a large effect on farm profitability. To understand the impact of selection for lowered methane emissions on the maternal ewe, genetic correlations between the characteristics for improved maternal production and methane emissions need to be estimated. The New Zealand Central Progeny Test (CPT) flock (McLean et al., 2006) has been measured for maternal or adult ewe traits. The CPT flocks are maintained to evaluate rams that have been selected for improving the maternal traits in their daughters, such as the number of lambs born, lamb survival and lamb growth. The initial progenitors of the divergent methane lines were selected from the CPT. The objective of this paper was therefore to use the large and comprehensive amount of maternal trait data that were collected in these research flocks to inform the likely trends and effects of selecting for reduced methane in the NZ commercial ewe population.

## Materials and methods

Animal experiments were conducted to meet the guidelines of the 1999 New Zealand Animal Welfare Act and were approved by the AgResearch Grasslands (Palmerston North, NZ) and AgResearch Invermay (Mosgiel, NZ) Animal ethics committees—application numbers 13282, 13604, 13742, 13743.

### Experimental animals

Trait data were available for animals from the five New Zealand recorded sheep flocks, born between 2007 and 2015, from the Sheep Improvement Limited (SIL) database (Newman et al., 2009). Three of the flocks consisted of the progeny of maternal dual-purpose sires generated in the New Zealand CPT program (SIL Flock IDs 4640, 4757 and 9153; McLean et al. (2006)). Sires within the CPT consisted of Coopworth, Romney, Perendale, Texel and Composite breeds, where the latter consisted primarily of combinations of the former breeds with additional infusions of Finn and East Friesian. All the CPT rams were mated to composite ewes. The fourth flock was the AgResearch research flock (SIL Flock ID 2638; Jonker et al. (2018)). The fifth flock was the methane yield selection line flock (SIL Flock ID 3633), which was established by screening the previously mentioned four flocks for methane yield, g CH_4_/kg DMI (Pinares-Patiño et al., 2013; Jonker et al., 2018). The high and low methane yield selection lines were created from the progeny of the top and bottom ten sires identified during the screening. The lines were closed to outside genetics in 2012 and all sires used from 2012 onwards were born in the methane yield selection flock. The lines are currently maintained at 100 ewes per line. From the 5 flocks, 2,206 animals born in 2007 and 2009–15 provided 9,333 methane yields for correlation with intestinal nematode disease traits (FEC1, NEM1, males and females) and adult ewe data collected from over 8,000 animals (Table 1). All animals were born and managed in a ryegrass-based pastoral grazing system.

**TABLE 1:** Years of birth, number of animals and data records for respiration chamber methane measures, disease and adult ewe maternal traits (at ages 2–6 years) by sex and birth flock.

Trait group	Descriptor	Birth flock^a^					
		2638	4640	4757	9153	3633	Total
Respiration chamber, females							
	Birth years	2009–11	2007, 09–13	2007, 09–11	2007, 09–11	2010–15	
	No. animals	480	460	159	132	442	1,673
	No. records	2,122	1,877	641	526	1,862	7,028
Respiration chamber, males							
	Birth years	2009				2010–15	
	No. animals	96				437	533
	No. records	375				1,930	2,305
Disease, females							
	Birth years	2007–15	2007–15	2007–12	2007–14	2012–14	
	No. animals	4,869	1,555	763	268	409	7,101
Disease, males							
	Birth years	2007–15	2007–12	2007–12	2007–11	2014–15	
	No. animals	5,029	657	678	230	286	6,202
Maternal, females^b^							
	Birth years	2007–15	2007–15	2007–15	2007–14	2010–15	
	No. animals	2,783	2,175	1,456	1,541	446	8,401
	No. records						
	LWMATE^b^, kg	7,795	6,709	1,057	3,133	1,102	19,796
	BCSMATE (1–5)	7,803	6,694	1,057	3,028	1,099	19,681
	PREGSC (0–3)	7,023	6,143	847	2,915	793	17,721
	NLB (0–3)	6,874	6,145	2,466	3,851	800	20,136
	LSW (0–1)	6,594	5,716	2,382	3,205	749	18,646
	FWMA, kg	5,728	4,039	74	151	786	10,778

Numbers in brackets are the range of scores possible under each maternal trait.

a 2638 AgResearch flock; 4640, 4757, 9153 Central Progeny Test (CPT) flocks; 3633 methane yield selection line flock.

b LWMATE, live weight at mating; BCSMATE, body condition score at mating; PREGSC, number of lambs pregnancy scanned; NLB, number of lambs born; LSW, litter survival to weaning; FWMA, fleece weight at mixed age.

### Maternal traits

Ewes, aged 2–6 years, had live weight (LW) and body condition score (BCS) recorded at mating, pregnancy scanning, lambing and weaning in most of the 5 flocks, together with pregnancy scanning data and fleece weight measured at the time of shearing. Of these traits, live weight at mating (LWMATE), body condition score at mating (BCSMATE), pregnancy scan number of lambs 0–3 (PREGSC), number of lambs born 0–3 (NLB) and fleece weight (mixed age, FWMA) were analysed. Litter survival to weaning (LSW) was calculated as the number of lambs weaned (0–3) divided by NLB. Body condition was scored 1–5 (in 0.5 units) by palpation to assess the amount of eye muscle and fat cover. Scores ranged from a 1 for emaciated to a 5 for obese (Suiter, 1994). Where possible, multiple measures per ewe were recorded to estimate the repeatability of measures over time.

### Methane measurements

Enteric methane emissions were measured in respiration chambers between 5 and 10 months of age (30–40 kg LW) as described by Pinares-Patiño et al. (2011, 2013). In addition, 66 ewes were measured as adults for 1–2 additional recording years to provide information for estimating the methane emission repeatabilities.

Animals were transitioned to a standard lucerne pellet diet (19% CP, 43% NDF and 10 MJ ME/kg dry matter) in pens over 21 days. This was followed by two measurement rounds (R1 and R2) of 2 days in respiration chambers, with rounds separated by a 10–15 day interval. Individual animal DMI was measured in metabolic crates during each round (4 days) and then in respiration chambers (2 days); the feeding level was based on LW and was estimated as twice their maintenance requirement, thus, variation in voluntary intake was not measured. Animals were fed two equal-sized meals at 9 a.m. (hour 0) and 3 p.m. (hour 6) daily. In both R1 and R2, individuals were randomly allocated to the four groups (each of 24 animals) and 24 respiration chambers, and typically 10 progeny per sire were randomly selected to be measured for CH_4_ emission. Only complete records were retained; additionally, if a chamber seal was broken or less than 95% of the offered feed was eaten on the day of measurement, then the record was discarded. The LW was measured during each methane measurement round. Here, three CH_4_ traits are reported, gross emissions expressed as g CH_4_/day (CH_4_/d), CH_4_ yield expressed as g CH_4_/kg DMI (CH_4_/DMI) and a derived measure of CH_4_ yield calculated from moles methane divided by moles of total gas emitted (i.e., moles CH_4_ plus moles of carbon dioxide) (CH_4_/(CH_4_+CO_2_)). The trait CH_4_/(CH_4_+CO_2_) was also computed in Jonker et al. (2018) in which it was shown to have a higher heritability and repeatability than CH_4_/DMI and can be a good indicator of CH_4_ yield where intake cannot be measured directly.

### Statistical analyses

Significant systematic effects and covariates were determined using a general linear model procedure (SAS, 2015). Table 2 gives full details of fixed effects and random effects fitted in the final model for each trait. Breed effects were not fitted explicitly, following Pickering et al. (2013) who found that breed accounted for very little variation in an animal model with similar populations.

**TABLE 2:** Effects in the final model for each trait.

Trait	Fixed effects^a^	Random effects^b^
CH_4_, g/d	byr.flk.sex, ryr.lot.group.round, brr, bdev	Animal, wgpe, agpe.ryr
CH_4_/DMI, CH_4_/(CH_4_+CO_2_)	byr.flk.sex, ryr.lot.group.round	Animal, wgpe, agpe.ryr
FEC1, NEM1	byr.flk.sex	Animal
LWMATE	rflk.ryr.mob.ageclass.2yo_RC, NLB_Prev, aod, brr	Animal, wgpe
BCSMATE	rflk.ryr.mob.ageclass.2yo_RC, NLB_Prev, brr	Animal, wgpe
PREGSC, NLB	rflk.ryr.mob.ageclass	Animal, wgpe
LSW	rflk.ryr.mob.ageclass, NLB	Animal, wgpe
FWMA	rflk.ryr.mob.ageclass, aod, brr	Animal, wgpe

a byr, birth year; flk, birth flock; ryr, recording year; lot, methane recording mob of 96 animals; group, sub-mob of up to 24 animals within a lot measured contemporaneously; round, methane recording measurement time 14 days apart; brr, birth rearing rank; bdev, birth day deviation; rflk, recording flock; ryr, recording year; mob, grazing mob; age class, age (years, 5 & 6 combined); 2yo_RC, respiration chamber for 2-year old record (0,1); NLB_Prev, NLB in (ryr -1); aod, age of dam; where “.” indicates an interaction.

b wgpe, within-group permanent environmental effect; agpe, across-group permanent environmental effect.

For methane traits, fixed class effects accounted for birth year * birth flock * sex combinations and for recording year * methane recording lot (mob of 96 animals) * group (sub-mob of up to 24 animals within a lot measured contemporaneously) * methane recording round combinations. In addition, a fixed effect for birth/rearing rank (brr) combining birth rank (single, twin, triplet) and rearing rank (single, twin, triplet), and a covariate for birth date deviation from the flock mean (bdev) were fitted for CH_4_/d. Transformation of FEC1 and NEM1 followed the current national breeding scheme (SIL) methodology in using log_e_ (x + 50), and birth year * birth flock * sex combinations were fitted.

Fixed effects fitted for each adult ewe trait were the age of dam (2–6+ years), birth/rearing rank and a contemporary group to account for recording flock * recording year * grazing mob * age class (2, 3, 4, 5 + 6 years) combinations. Animals that were measured in the first year of life in respiration chambers at AgResearch Grasslands (Palmerston North, NZ) had been transported 1,130 km from where they were grazed for approximately 1 month. Additional levels were added to the contemporary group effects for the 2-year-old LWMATE and BCSMATE to differentiate these management groups. An additional fixed effect was fitted for LWMATE and BCSMATE at 3 years of age and older to account for the number of lambs born in the previous recording year (NLB_Prev).

Pedigree records (individual, sire and dam) were available for 81,936 animals born between 1990 and 2015 and were used to construct genetic relationships for fitting a general animal model in ASREML (Gilmour et al., 2009).

### Genetic parameters

Methane traits were fitted into a linear mixed model (1). Three random effects were fitted with normal distributions. These were individual additive genetic effects, across and within round permanent environmental effects.

$$y=\mathrm{Xb}+\mathrm{Za}+W_{r}pe_{w}+W_{y}pe_{y}+e$$ (1)

where **y** is the vector of observations, **b** is the vector of fixed effects, **a** is the vector or random animal genetic effect, **pe**_**w**_ is the vector of permanent environment effect within methane measurement round (i.e., day 1 and day 2), **pe**_y_ is the vector of permanent environmental effect and non-additive genetic effects across methane measurement rounds (round 1 and round 2), **e** is the vector of random residual effects, **X** is the design matrix of full column rank that associates observations with the appropriate combination of fixed effects, **Z** is the design matrix that associates observations with the appropriate combination of random effects, **W**_**w**_ and **W**_**r**_ are design matrices relating observations to animals within and across methane measurement rounds, respectively, and **A** is the numerator relationship matrix based on pedigree information, var (**pe**) = **I**σ^2^_pe_, var (**e**) = **I**σ^2^_e_ and var (**a**) = **A**σ^2^_e_.

The phenotypic correlation between records is equal to the repeatability. Within the animal, the “repeatability” of trait measures was estimated using the intra-class correlation—the ratio between individual variance (additive genetic plus permanent environmental effects) and the phenotypic variance.

Single trait and bivariate (two-trait) analyses were undertaken using ASREML 3.0 (Gilmour et al., 2009), and phenotypic, genetic and environmental correlations between the traits were estimated.

## Results

Phenotypic means, standard deviations and heritability estimates for methane traits, internal parasites and maternal traits are detailed in Table 3. Repeatability estimates from measures in multiple recording years are described in Table 3 for methane and maternal traits. Table 4 gives phenotypic and genetic correlations between methane, FEC1, NEM1 and maternal traits.

**TABLE 3:** Means, standard deviations, heritability (h^2^) and repeatability estimates across recording years (±SE) for respiration chamber methane traits, disease and maternal traits.

Trait	No. animals	No. records	Mean	STD	σ_p_	h^2^ (SE)	Repeatability (SE)
Respiration chamber							
CH_4_, g/d	2,206	9,333	24.0	4.40	2.99	0.26 (0.03)	0.42 (0.05)
CH_4_/DMI, g/kg	2,206	9,332	15.4	2.10	1.48	0.12 (0.02)	0.20 (0.03)
CH_4_/(CH_4_+CO_2_), mol/mol	2,206	9,006	0.059	0.006	0.005	0.21 (0.03)	0.34 (0.06)
Disease: Faecal egg counts (log_e_ (value + 50))							
FEC1	14,744		6.07	0.90	0.79	0.33 (0.02)	
NEM1	13,815		4.30	0.59	0.55	0.30 (0.02)	
Maternal							
LWMATE, kg	6,900	19,796	67.3	8.27	6.78	0.49 (0.02)	0.71 (0.01)
BCSMATE (1–5)	6,888	19,681	3.57	0.69	0.49	0.20 (0.02)	0.28 (0.01)
PREGSC (0–3)	6,649	17,721	1.94	0.73	0.72	0.08 (0.01)	0.12 (0.01)
NLB (0–3)	7,919	20,136	1.91	0.73	0.71	0.08 (0.01)	0.12 (0.01)
LSW (0–1)	7,763	18,646	0.78	0.35	0.34	0.05 (0.01)	0.11 (0.01)
FWMA, kg	4,467	10,778	4.13	0.92	0.66	0.59 (0.03)	0.71 (0.01)

**TABLE 4:** Genetic (*r*_g_) and phenotypic (*r*_p_) correlations (±SE) between respiration chamber methane emissions (CH_4_, g/d), methane yield traits (CH_4_/DMI, g/kg) and (CH_4_/(CH_4_+CO_2_), mol/mol) on a restricted diet, FEC1, NEM1 and maternal traits. Correlations significant at the 5% level (*p* < 0.05) are marked with “*”.

	CH_4_, g/d		CH_4_/DMI, g/kg		CH_4_/(CH_4_ + CO_2_), mol/mol	
	*r*_g_	*r*_p_	*r*_g_	*r*_p_	*r*_g_	*r*_p_
FEC1	0.18 (0.09)	0.09 (0.03)*	0.13 (0.11)	0.05 (0.02)*	0.14 (0.10)	0.04 (0.02)
NEM1	0.01 (0.10)	0.03 (0.03)	0.03 (0.11)	0.02 (0.02)	0.03 (0.11)	0.03 (0.03)
LWMATE	0.77 (0.05)*	0.41 (0.02)*	0.31 (0.09)*	0.06 (0.02)*	0.28 (0.09)*	0.07 (0.02)*
BCSMATE	0.35 (0.09)*	0.12 (0.02)*	0.22 (0.11)*	0.003 (0.02)	0.21 (0.10)*	0.05 (0.02)*
PREGSC	−0.05 (0.12)	0.01 (0.02)	0.11 (0.13)	−0.02 (0.01)	0.05 (0.13)	−0.02 (0.02)
NLB	−0.07 (0.12)	0.02 (0.02)	0.09 (0.13)	−0.01 (0.01)	0.05 (0.12)	−0.02 (0.02)
LSW	−0.20 (0.14)	−0.01 (0.02)	−0.13 (0.15)	0.01 (0.01)	−0.13 (0.15)	0.03 (0.02)
FWMA	−0.10 (0.08)	−0.01 (0.02)	−0.21 (0.10)*	−0.07 (0.02)*	−0.21 (0.09)*	−0.08 (0.02)*

### Heritability estimates

Heritability (and repeatability) estimates were high for LWMATE 0.49 ± 0.02 (0.71 ± 0.01) and for fleece weight 0.59 ± 0.03 (0.71 ± 0.01) and moderate for BCSMATE 0.20 ± 0.02 (0.28 ± 0.01). The heritability (and repeatability) estimates for the NLB, LSW and pregnancy scanning data were lower at ∼0.1; however, these estimates are similar to the current national estimates derived from the SIL methodology. The heritability estimates for CH_4_/d (0.26 ± 0.03) and CH_4_/DMI (0.12 ± 0.02) were similar to previous estimates using this population (Pinares-Patiño et al., 2013). The heritability of methane yield derived for the ratio of methane emissions to total gas emissions (CH_4_/(CH_4_ + CO_2_)) at 0.21 ± 0.03 (repeatability 0.34 ± 0.06) was higher than the methane yield derived for the ratio of methane emissions to dry matter intake (CH_4_/DMI) at 0.12 ± 0.02 (repeatability 0.20 ± 0.03). For single measures of FEC1 and NEM1, the heritability estimates were 0.33 ± 0.02 and 0.30 ± 0.02, respectively.

### Correlations

Fleece weight had a negative correlation with methane yield traits (−0.21 ± 0.10 for CH_4_/DMI); LW and BCS had positive genetic correlations with methane yield traits (0.31 ± 0.09, 0.22 ± 0.11, respectively for CH_4_/DMI). Given the growing body of evidence for a link between increased lean mass associated with low methane, monitoring of these traits should continue.

For FEC1 and NEM1, there were no significant genetic correlations with methane gross emissions or yield traits; phenotypic correlations for FEC1 were significant but low for methane gross emissions and CH_4_/DMI (0.09 ± 0.03, 0.05 ± 0.02, respectively).

## Discussion

If breeding is used as a mitigation approach for the reduction of enteric methane emissions for sheep, the most substantial reduction would come from the lower emissions in adult ewe flocks. Methane measures, however, have been largely collected in young animals. Genetic correlations between lamb and ewe measurements of CH_4_ and CO_2_, using portable accumulation chambers, ranging from 0.85 to 0.99 (Jonker et al., 2018). This provides evidence that methane can be measured in young females to predict methane production later in life. The impact of breeding for lowered methane emissions on important maternal traits has not been evaluated to date, because lifetime performance records in large flocks measured for methane emissions have been extremely scarce. This is largely due to the need to maintain and monitor large flocks of sheep through multiple pregnancies. Here, we have collated an unprecedented data set, including thousands of records from research flocks recorded over many years, to investigate the relationship between methane traits measured in young animals and maternal ewe traits. Results suggest that breeding for low methane yield is unlikely to have a substantial negative impact on important adult ewe traits such as the ability to maintain weight and condition, fecundity and parasite resistance.

For methane yield traits (CH_4_/DMI and CH_4_/(CH_4_+CO_2_)), there were no genetic correlations with maternal fertility, and litter survival traits were significantly different from zero, suggesting that selection for low methane yield animals is unlikely to have any negative effects on the commercial ewe flocks.

Pregnancy scanning data, NLB and lamb survival to weaning did not appear to have any relationship with methane traits. This indicates that selection for low methane yield is unlikely to affect these traits. FEC1 and NEM1 were not genetically correlated with methane traits although a low phenotypic correlation with FEC1 was observed. This phenotypic relationship is unsurprising as a parasitized animal is likely to reduce intake and emit less methane. These results do not reflect the increased methane yield found by Fox et al. (2018), who challenged 24 sheep with infective Teladorsagia circumcinta larvae. This is likely due to additional power from the sample size in the current study, where over 13,000 FEC records and over 9,000 methane records have been used to estimate the genetic correlation between parasite burden and methane emissions in grazing sheep under natural challenges.

Low to moderate relationships were observed between methane yield, fleece weight, body weight and body condition score at mating. It has been shown previously that low-emitting animals tend to have a greater amount of lean tissue (Elmes et al., 2014), possibly due to greater ratios of propionate and valerate to acetate and butyrate coming from the rumen (Jonker et al., 2019). Also, Bilton et al. (2021) showed that low methane ewes similarly had different volatile fatty acid profiles in their rumen fluid and less short-chain fatty acids in their milk. Although there is no evidence here that these relationships affect fecundity, a watching brief should be maintained for traits associated with fat deposition, such as BCS traits and potentially for puberty, which has a known association with growth and body composition (Stephenson et al., 1980; Rosales Nieto et al., 2014).

Fleece weight has previously been reported as having a significant negative correlation with methane yield (Pinares-Patiño et al., 2013). This is an interesting relationship as the expectation would be that a bigger ewe would have a heavier fleece (Wuliji et al., 2011), and increased fleece weight, given a set body weight, is associated with increased feed intake during *ad libitum* feeding (Schinckel, 1960). The underpinning mechanism for increased wool growth associated with lowered methane yield is unknown, but it reflects that selection for changing methane emissions and the resulting impact on rumen outflow may have effects on the animal that are yet to be understood.

An important caveat is that selection based on fixed intake in respiration chambers may not reflect true methane yield on pasture, where intake varies based on availability and quality of feed. Jonker et al. (2018) have reported genetic correlations ranging between 0.4 and 0.7 for different methane measurements on the same animals through respiration chambers and later through portable accumulation chambers. This infers that additional evidence for the relationship between methane emissions, feed intake and production traits is still required.

Further work has been documented using data from portable accumulation chambers, estimating methane yield by the ratio of moles of methane to moles of total gas in the absence of intake data but where voluntary intake is allowed to vary (Goopy et al., 2011; Robinson et al., 2015; Goopy et al., 2016; Paganoni et al., 2017; Jonker et al., 2018). The estimated genetic correlation between CH_4_/(CH_4_ + CO_2_) and CH_4_/DMI is very high (rg = 0.94 ± 0.03) and CH_4_/(CH_4_ + CO_2_) is more heritable and repeatable. The correlations between the two measures of methane yield and adult ewe traits were also very similar. This suggests that at a fixed rate of intake, gas trait measures can be used as a proxy for intake. The advantages of portable accumulation chamber measures are manifold: each measure is approximately ten-fold lower in cost and animals can be measured at pasture and at any stage of life, e.g., during pregnancy. As more data are collected and error rates are minimized, better estimates of the relationship between methane traits and maternal traits should emerge.

## Conclusion

Results suggest that breeding for low-methane yield is unlikely to negatively affect fecundity or parasite resistance in adult ewes. Low to moderate genetic correlations were seen for methane yield with wool growth, LW, and BCS. Fleece weight was negatively correlated with methane yield, and LW and BCS were positively correlated with methane yield. For the methane yield traits, the heritability and repeatability of CH_4_/(CH_4_+CO_2_) were higher than CH_4_/DMI suggesting that gas traits are an efficient and effective proxy for DMI at a fixed level of feed intake. This suggests that opportunities exist for the use of alternative methods, such as portable accumulation chambers, to attain a greater number of measures of methane emissions from grazing sheep at different reproductive stages. Finally, breeding is a suitable mitigation strategy for lowering ruminant methane; a watching brief is needed to ascertain the long-term impacts of the associated changing rumen outflow with body weight and BCS. Our results are relevant to other ruminant species and in the assessment of other mitigation technologies.

## Data Availability

The original contributions presented in the study are included in the article; further inquiries can be directed to the corresponding author.

## References

[B1] Bain W. E. Bezuidenhout L. Jopson N. B. Pinares-Patiño C. McEwan J. C. (2014). Rumen differences between sheep identified as being low or high methane emitters. Proc. Assoc. Advmt. Anim. Breed. Genet. 20, 376–378.

[B2] Beef and Lamb NZ Economic reports (2020). Stock number survey. Available at: https://beeflambnz.com/sites/default/files/data/files/Stock-Number-Survey-30-June-2020.pdf.

[B3] Bilton T. P. Hickey S. M. Janssen P. H. Jonker A. J. Hess M. K. Bryson B. (2021). Impact of breeding for divergent methane yield on milk composition in breeding ewes. Proc. Assoc. Advmt. Anim. Breed. Genet. 24, 15–18.

[B4] Borg R. C. Notter D. R. Kott R. W. (2009). Genetic analysis of Ewe stayability and its association with lamb growth and adult production. J. Anim. Sci. 87, 3515–3524. 10.2527/jas.2008-1623 19648486

[B5] Elmes S. N. Bain W. E. Greer G. J. Hickey S. M. Young E. A. Pickering N. K. (2014). An exploratory investigation of the effects of selection for divergence in methane emissions on rumen digesta and carcass traits in eight-month-old sheep. Proc. N. Z. Soc. Ani. Prod. 74, 142–144.

[B6] Everett-Hincks J. M. Dodds K. G. (2008). Management of maternal-offspring behavior to improve lamb survival in easy care sheep systems. J. Anim. Sci. 86, E259–E270. 10.2527/jas.2007-0503 17965331

[B7] Fox N. J. Smith L. A. Houdijk J. G. M. Athanasiadou S. Hutchings M. R. (2018). Ubiquitous parasites drive a 33% increase in methane yield from livestock. Int. J. Parasitol. 48 (13), 1017–1021. Epub 2018 Aug 11. PMID: 30107148. 10.1016/j.ijpara.2018.06.001 30107148

[B8] Gilmour A. R. Gogel B. J. Cullis B. R. Thompson R. (2009). ASReml UserGuide release 3.0. UK: VSN International Ltd, Hemel Hempstead, HP1 1ES.

[B9] Golub A. Henderson B. B. Hertel T. W. Gerber P. J. Rose S. K. Sohngen B. (2013). Global climate policy impacts on livestock, land use, livelihoods, and food security. Proc. Natl. Acad. Sci. U. S. A. 110, 20894–20899. 10.1073/pnas.1108772109 23019587 PMC3876243

[B10] Goopy J. P. Chang C. Tomkins N. (2016). “A comparison of methodologies for measuring methane emissions from ruminants,” in Methods for measuring greenhouse gas balances and evaluating mitigation options in smallholder agriculture. Editors Rosenstock T. S. Rufino M. C. Butterbach-Bahl K. Wollenberg L. Richards M. (Springer International Publishing). 10.1007/978-3-319-29794-1_5

[B11] Goopy J. P. Donaldson A. Hegarty R. S. Vercoe P. E. Haynes F. Barnett M. (2013). Low methane yield sheep have smaller rumens and shorter rumen retention time. Br. J. Nutr. 8, 578–585. 10.1017/S0007114513002936 24103253

[B12] Goopy J. P. Donaldson A. Hegarty R. S. Vercoe P. E. Haynes F. Barnett M. (2014). Low-methane yield sheep have smaller rumens and shorter rumen retention time. Br. J. Nutr. 111, 578–585. 10.1017/S0007114513002936 24103253

[B13] Goopy J. P. Robinson D. L. Woodgate R. S. Donaldson A. Oddy V. H. Vercoe P. E. (2015). Estimates of repeatability and heritability of methane production in sheep using portable accumulation chambers. Anim. Prod. Sci. 56, 116. 10.1071/AN13370

[B14] Goopy J. P. Woodgate R. Donaldson A. Robinson D. L. Hegarty R. S. (2011). Validation of a short-term methane measurement using portable static chambers to estimate daily methane production in sheep. Anim. Feed Sci. Technol. 166–167, 219–226. 10.1016/j.anifeedsci.2011.04.012

[B15] Hess M. K. Donaldson A. Henry H. M. Robinson D. L. Hess A. S. McEwan J. C. (2020). Across-country prediction of methane emissions using microbial profiles. Proc. Assoc. Advmt. Anim. Breed. Genet. 24, 163–166.

[B16] Jonker A. Hickey S. M. Janssen P. H. Shackell G. Elmes S. Bain W. E. (2018). Genetic parameters of methane emissions determined using portable accumulation chambers in lambs and ewes grazing pasture and genetic correlations with emissions determined in respiration chambers. J. Anim. Sci. 96, 3031–3042. 10.1093/jas/sky187 29741677 PMC6095386

[B17] Jonker A. Hickey S. M. McEwan J. C. Rowe S. J. Janssen P. H. MacLean S. (2019). Genetic parameters of plasma and ruminal volatile fatty acids in sheep fed alfalfa pellets and genetic correlations with enteric methane emissions1. J. Anim. Sci. 97, 2711–2724. 10.1093/jas/skz162 31212318 PMC6606511

[B18] Kittelmann S. Pinares-Patiño C. S. Seedorf H. Kirk M. R. Ganesh S. McEwan J. C. (2014). Two different bacterial community types are linked with the low-methane emission trait in sheep. PLoS One 9, e103171. 10.1371/journal.pone.0103171 25078564 PMC4117531

[B19] McLean N. J. Jopson N. B. Campbell A. W. Knowler K. Behrent M. Cruickshank G. (2006). An evaluation of sheep meat genetics in New Zealand: The central progeny test CPT. Proc. N. Z. Soc. Ani. Prod. 66, 368–372.

[B20] Ministry for the Environment (MfE) (2021). New Zealand’s greenhouse gas inventory 1990-2019. An overview. Available at: www.mfe.govt.nz (Accessed May 7, 2021).

[B21] Morgan-Davies C. Waterhouse A. Pollock M. L. Milner J. M. (2007). Body condition score as an indicator of Ewe survival under extensive conditions. Anim. Welf. 17 (1), 71.

[B22] Newman S. A. N. McEwan J. C. Young M. J. (2009). A decade of sheep improvement limited (SIL). Proc. Assoc. Advmt. Anim. Breed. Genet. 18, 624–627.

[B23] Paganoni B. Rose G. Macleay C. Jones C. Brown D. J. Kearney G. (2017). More feed efficient sheep produce less methane and carbon dioxide when eating high-quality pellets. J. Anim. Sci. 95 (9), 3839–3850. 10.2527/jas2017.1499 28992015

[B24] UNFCCC (2015). Paris agreement to the united nations framework convention on climate change. T.I.A.S. No. 16–1104.

[B25] Pickering N. K. Blair H. T. Hickson R. E. Dodds K. G. Johnson P. L. McEwan J. C. (2013). Genetic relationships between dagginess, breech bareness, and wool traits in New Zealand dual-purpose sheep. J. Anim. Sci. 91, 4578–4588. 10.2527/jas.2013-6741 23893990

[B26] Pinares-Patiño C. S. Ebrahimi S. H. McEwan J. C. Dodds K. G. Clark H. Luo D. (2011). Is rumen retention time implicated in sheep differences in methane emission? Proc. N. Z. Soc. Ani. Prod. 71, 219–222.

[B27] Pinares-Patiño C. S. Hickey S. M. Young E. A. Dodds K. G. MacLean S. Molano G. (2013). Heritability estimates of methane emissions from sheep. Animal 7 (2), 316–321. 10.1017/S1751731113000864 PMC3691003 23739473

[B28] Robinson D. L. Goopy J. P. Hegarty R. S. Oddy V. H. (2015). Comparison of repeated measurements of methane production in sheep over 5 years and a range of measurement protocols. J. Anim. Sci. 93, 4637–4650. 10.2527/jas.2015-9092 26523556

[B29] Rosales Nieto C. A. Thompson A. N. Macleay C. A. Briegel J. R. Hedger M. P. Ferguson M. B. (2014). Relationships among body composition, circulating concentrations of leptin and follistatin, and the onset of puberty and fertility in young female sheep. Anim. Reprod. Sci. 151 (3), 148–156. 10.1016/j.anireprosci.2014.10.008 25458319

[B30] Rowe S. J. Hickey S. M. Johnson P. L. Bilton T. P. Jonker A. Bain W. (2021). The contribution animal breeding can make to industry carbon neutrality goals. Proc. Assoc. Advmt. Anim. Breed. Genet. 24 (15), 18.

[B31] Rowe S. J. Hickey S. M. Jonker A. Hess M. K. Janssen P. Johnson P. L. (2019). Selection for divergent methane yield in New Zealand sheep – A ten-year perspective. Proc. Assoc. Advmt. Anim. Breed. Genet. 23, 306–309.

[B37] SAS (2015). Base SAS 9.4 Procedures Guide. 5th Edn. Cary, NC: SAS Institute Inc..

[B32] Schinckel P. J. (1960). Variation in feed intake as a cause of variation in wool production of grazing sheep. Aust. J. Agric. Res. 11, 585–594. 10.1071/ar9600585

[B33] Springmann M. Mason-D’Croz D. Robinson S. Wiebe K. Charles H. Godfray H. J. (2016). Mitigation potential and global health impacts from emissions pricing of food commodities. Nat. Clim. Chang. 7, 69–74. 10.1038/nclimate3155

[B34] Stephenson S. K. Dalton D. C. Kirton A. H. (1980). The relationships of growth, body shape, and body composition to the initiation of oestrous activity in different sheep breeds. Proc. N. Z. Soc. Ani. Prod. 40, 258–267.

[B35] Suiter R. J. (1994). Body condition scoring of sheep and goats. Perth: Farmnote 69–1994, Western Australian Department of Agriculture.

[B36] Wuliji T. Dodds K. G. Andrews R. N. Turner P. R. (2011). Selection response to fleece weight, wool characteristics, and heritability estimates in yearling Romney sheep. Livest. Sci. 134, 26–31. 10.1016/j.livsci.2010.06.003

